# Photo Quiz: Persistent dysuria and hematuria in a 9-year-old male

**DOI:** 10.1128/jcm.01371-25

**Published:** 2026-02-11

**Authors:** Heather L. Young, Rachel A. Frenner, Bobby L. Boyanton

**Affiliations:** 1 Department of Pediatrics, College of Medicine, Section of Pediatric Infectious Diseases, University of Arkansas for Medical Sciences and Arkansas Children’s Hospital 12215, Little Rock, Arkansas, USA; 2 Department of Pathology and Laboratory Medicine, Arkansas Children's Hospital 14423, Little Rock, Arkansas, USA; 3 Department of Pathology and Laboratory Medicine, College of Medicine, University of Arkansas for Medical Sciences 155638, Little Rock, Arkansas, USA; Mayo Clinic Minnesota, Rochester, Minnesota, USA

## PHOTO QUIZ 

**Fig 1:**
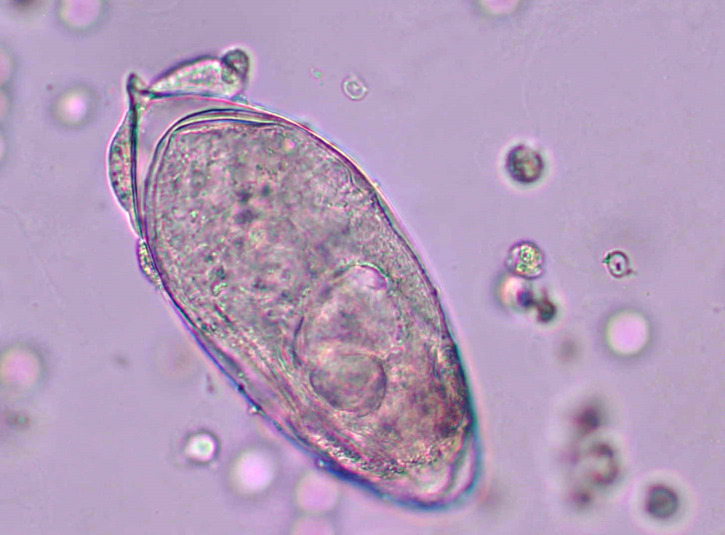
Unstained microscopic image of urine specimen following centrifugation (magnification, 400×).

A 9-year-old male presented to the emergency department with a 4-month history of painful urination and bright-red urine. He denied fever, chills, fatigue, nausea, emesis, diarrhea, and abdominal pain. Despite urine cultures being negative, his community healthcare provider and an outside hospital had prescribed antibiotics three separate times for suspected urinary tract infection. He immigrated from Mali (West Africa) 1 year prior. He was afebrile, and the physical examination was normal; pertinent negatives included absence of genitourinary abnormalities/pain and palpation-induced costovertebral angle pain. Complete blood count showed a white blood count of 6.59 10^9^/L (normal: 4.50–13.50), 50% lymphocytes, 27% neutrophils, 15% eosinophils; hemoglobin of 138 g/L (normal: 115–155); platelet count of 369 10^9^/L (normal: 150–400). Urinalysis was significant for moderate blood (normal, negative), negative nitrite (normal, negative), small leukocytes (normal, negative), and total protein of 1 g/L (normal, negative). Urine microscopic examination was significant for 391 leukocytes/high-power-field (normal: <5), 245 red blood cells/high-power-field (normal, <3), and the organism depicted in Fig. 1. Urine culture was negative. Retroperitoneal ultrasound disclosed normal kidneys and ureters; the bladder was mildly distended.

## ANSWER TO PHOTO QUIZ

The patient was diagnosed with urinary schistosomiasis due to *Schistosoma haematobium* and promptly seen by a pediatric infectious disease specialist. Schistosoma serum IgG antibody was positive. Additional infectious disease tests were all negative, including stool ova and parasites, human immunodeficiency virus 1 & 2 screening (p24 antigen + HIV-1/2 antibody), hepatitis (A–C), and tuberculosis (T-SPOT TB, Revvity, formerly Oxford Immunotec). Following praziquantel treatment, repeat urinalysis was normal and he was no longer experiencing dysuria or gross hematuria.

Schistosomiasis is a blood fluke (*Schistosoma* trematode worm) parasitic disease affecting the intestinal or urogenital tracts (1). It is primarily observed in tropical and subtropical areas, especially in communities lacking adequate sanitation and safe drinking water. Although being reported in more than 70 countries, approximately 90% of cases occur in Africa (1). Transmission requires an egg-contaminated water source, an intermediary host (freshwater snail), and human contact with water inhabited by the intermediary host (2). Cercariae released from the intermediary host penetrate human skin, enter the circulation, and based upon the *Schistosoma* species, develop into adult worms that inhabit the mesenteric vessels of the intestinal or urogenital tracts. Mature female worms lay eggs that migrate into the stool or bladder in cases of intestinal or genitourinary schistosomiasis, respectively (2). Intestinal schistosomiasis, not discussed further, is caused by the following *Schistosoma* species: S. *mansoni, S. japonicum, S. mekongi, S. guineensis,* and *S. intercalatum*. Urogenital schistosomiasis due to *S. haematobium* is restricted geographically to Africa and the Middle East, while *S. haematobium-S. bovis* hybrids predominate in Corsican France (1, 3). Acute urogenital schistosomiasis usually presents with an itchy maculopapular rash and/or hematuria, the latter of which was present in our patient (2). Left untreated, damage and fibrosis will occur to the bladder, kidney, and ureter and may extend to reproductive organs leading to male and female infertility (1, 2). In long-standing cases, prolonged chronic inflammation can lead to malignancy (4).

The diagnosis of urinary schistosomiasis is challenging early in the disease process and relies heavily on the physical examination and clinical/travel history in conjunction with abnormal urinalysis findings (e.g., proteinuria and microscopic hematuria). The eggs of *S. haematobium* will not be observed in a concentrated urine specimen until at least 90 days following cercarial infection (5). When present, the eggs are oval (110–170 µm × 40–70 µm) with a terminal spine and contain a single miracidium (5). Other diagnostic testing, including antigen, antibody, and polymerase chain reaction (direct specimen and cell-free DNA), is available and reviewed elsewhere (5). In our case, *Schistosoma* IgG antibody testing was ordered prior to urine microscopic results being available to the treating physician. This highlights noteworthy items for discussion. From a test utilization standpoint, electronic health record systems need artificial intelligence-based solutions to help curtail unnecessary laboratory testing as human-based solutions are unreliable and not practical. From a test performance perspective, three major points must be considered. First, *Schistosoma* antibody testing is non-specific and serologic cross-reactivity with other helminth infections can yield a false-positive result (5). Second, a positive *Schistosoma* serologic test result cannot distinguish chronic from past infection. Third, *Schistosoma* serologic test results may be negative during acute infection, or in chronically infected patients with low organism burden (5).

As highlighted in our case, obtaining immigration and/or travel history is a critical component of a healthcare provider’s diagnostic arsenal. Eliciting such history would have led to earlier diagnosis and treatment of this pediatric patient with schistosomiasis.
